# Forced expression of heat-shock protein 70 increases the secretion of Hsp70 and provides protection against tumour growth

**DOI:** 10.1038/sj.bjc.6601583

**Published:** 2004-02-17

**Authors:** M-H Wang, M E Grossmann, C Y F Young

**Affiliations:** 1 Department of Urology and Biochemistry/Molecular Biology, Mayo Graduate School, Mayo Clinic/Foundation, Rochester, MN 55905, USA

**Keywords:** heat-shock protein 70, secretion, prostate cancer, transgenic adenocarcinoma mouse prostate, gene therapy

## Abstract

Although heat-shock protein 70 (Hsp70) has been considered an intracellular protein, we report that Hsp70 is secreted under normal cell culture conditions by human prostate cell lines, LAPC-4, PC-3, CWR-22, RWPE-1 and -2, LNCaP, and TRAMP (transgenic adenocarcinoma mouse prostate)-C2. We found that the secretion can be enhanced by transfection with cDNA encoding for Hsp70. To verify that the Hsp70 detected in the supernatant was not secondary to cell leakage, C2 cells were cotransfected with cytoplasmic *Renilla luciferase* as a reporter. High levels of activities were noted in the cell extracts, while no enzyme activities were detected in the supernatants. To verify that forced oversecretion of Hsp70 could protect against tumour growth, mice were injected with C2 cells transfected with an Hsp70 DNA construct and challenged with live tumour cells. Mice injected with cells transfected with the Hsp70 DNA construct demonstrated a significantly decreased rate of tumour growth compared to those injected with empty vector. In addition, a difference in survival rate as defined by a surrogate end point was noted between the two groups. In a second experiment, we developed a cell line that stably overexpressed Hsp70. Mice injected with these cells also demonstrated a significant decrease in tumour growth and significantly increased survival.

Prostate cancer is the second leading cause of cancer death in men in the United States, exceeded only by lung cancer. The American Cancer Society estimates that 28 900 men in the United States will die of prostate cancer in the year 2003. Currently, for patients with early stage, organ confined disease, there are well-defined treatment options, including radical prostatectomy, radiation therapy, or watchful waiting. However, no definitive treatments are available for advanced or recurrent disease. It is known that tumour regression can be achieved with androgen blockade; however, disease usually recurs within 1–2 years, leading to significant morbidity and mortality (Mahler and Denis, 1992).

The idea of gene transfer to enable the use of host immune system against tumours has generated new treatment options for patients with prostate cancer. This therapy is based on the assumption that it is possible to break tolerance to tumour antigens by increased expression of immunomodulants and chemokines (Houghton, 1994). Specifically in prostate cancer studies, various gene transfer strategies using human or murine granulocyte/macrophage colony-stimulating factor (Sanda *et al*, 1994), interleukin-2 (Fearon *et al*, 1990), and interferon gamma (Vieweg *et al*, 1994) have been shown to elicit antitumour responses.

The antitumour property of heat-shock proteins (hsp's) was recognised in the 1980s when purified hsp's from tumour cells were shown to elicit immunity (Srivastava *et al*, 1998). Subsequent studies have contributed to the understanding of the mechanism by which purified hsp's interact with the immune system. In their role as housekeeping proteins and chaperones, hsp's can bind to multiple intracellular peptides including tumour peptides (Gething and Sambrook, 1992; Parsell and Lindquist, 1993). These hsp–peptide complexes have the unique ability to promote crosspriming of cytotoxic T lymphocytes (CTLs), one of the most effective ways to stimulate antitumour immunity (Cavallo *et al*, 1993; Suto and Srivastava, 1995; Cayeux *et al*, 1997). Once released from tumour cells, these hsp complexes bind to CD91 receptors on host antigen-presenting cells (APCs) (Binder *et al*, 2000). The binding of hsp–peptide complex with CD91 leads to the internalisation of the complex and presentation of tumour peptides with MHC class I with the activation of CD8+ cells. Studies also suggest that a small proportion of the hsp–peptide complex is loaded onto MHC class II, leading to the stimulation of CD4+ cells (Matsutake and Srivastava, 1999). Other receptors, such as CD36 and CD40, were identified recently on APCs that can also interact with hsp's (Panjwani *et al*, 2000; Wang, 2001). These receptors, once activated, cause the secretion of nonspecific inflammatory cytokines such as tumour necrosis factors and interleukins (Ishii *et al*, 1999).

Recent studies indicated that hsp's interact with natural killer (NK) cells. Studies have demonstrated a correlation between tumour cell hsp's expression and increased NK cell-mediated cell lysis (Ponomarev *et al*, 2000). This observation is further supported by the identification of the extracellular C-terminal epitopes on Hsp70, 504-617, which are important for NK cells' killing activities (Botzler *et al*, 1998).

Previously in our laboratory, we found that purified Hsp70 from transgenic adenocarcinoma mouse prostate (TRAMP)-C2 cells (Foster *et al*, 1997) can induce an antitumour response (Vanaja *et al*, 2000). TRAMP-C2 is a murine prostate cancer cell line derived from TRAMP mice (transgenic adenocarcinoma of mouse prostate) that spontaneously develop prostate cancer (Greenberg *et al*, 1995). In the process of extending our previous studies on hsp's, we noted the presence of Hsp70 in the routine cell culture media of prostate cancer cells. This led us to investigate Hsp70 secretion and its significance in antitumour therapy. The advantage of using hsp's gene transfer is that it bypasses the need to purify large quantities of hsp's, in addition to allowing for systemic delivery.

## MATERIALS AND METHODS

### Cell lines

TRAMP C2 cells were cultured in Dulbecco's modified Eagle's medium (DMEM, GIBCO, CA, USA) supplemented with 5% fetal calf serum (FCS), and 1% penicillin/streptomycin. Cells were maintained in T162 cm flasks at 37°C, 5% CO_2_, and passaged weekly. Cells used for animal injections were collected by trypsinisation and washed with DMEM three times prior to injections. Specified number of viable cells (100 *μ*l of DMEM per mouse) was determined by trypan blue exclusion, and used for injection.

LNCaP, PC-3, CWR-22, and LAPC-4 are human prostate adenocarcinoma cell lines. Each cell line was cultured in RPMI 1640 media (GIBCO, CA, USA) containing 5% FCS and 1% penicillin/streptomycin. Cells were maintained at 37°C, 5% CO_2_. RWPE-1 and RWPE-2 cells derived from normal human prostate cells immortalised with human papilloma virus 18 were maintained in keratinocyte media (GIBCO, CA, USA), 37°C, and 5% CO_2_ (Bello *et al*, 1997).

### Hsp70-expressing cells

The full-length cDNA coding for inducible mouse Hsp70 was inserted into a mammalian expression vector pcDNA3.1 (+) (Invitrogen, CA, USA), and transiently transfected into TRAMP-C2 cells as per the protocol (Superfect, Qiagen, CA, USA). Empty vector was transfected as a control. Supernatants and cell extracts were collected at 24, 48, and 72 h. Whole-cell extracts were prepared as per Santa Cruz Biotechnology research applications. Spent media were spun down at 1000 r.p.m. for 5 min; supernatant was collected and concentrated with Vivaspin column concentrator, 10 000 MWCO (Vivascience, CA, USA). Protein levels were quantified with DC protein assay or Bradford (Bio-Rad, CA, USA). Success of the transfections was verified by Western analysis for Hsp70. To generate stable clones, cells were transfected with pcDNA3.1+Hsp70 or empty vector as above and selected with gentamicin. Positive clones were selected and verified by Western analysis for Hsp70.

### Secretion study

TRAMP-C2 cells were cotransfected with pcDNA3.1+Hsp70. and *Renilla luciferase* vector (Promega, WI, USA). Supernatants and cell extracts were collected at 24, 48, and 72 h. Proteins were collected and analysed by Western analysis for Hsp70 as described below and luciferase activity was measured as per the manufacturer's instructions (Promega, WI, USA). Western blots of Hsp70 protein were quantified by densitometry and luciferase activity was measured by luminescence. All experiments were performed in triplicate.

LNCaP cells were cultured in RPMI 1640 media (GIBCO, CA, USA) containing 5% FCS, 1% penicillin/streptomycin, and 1 nM mibolerone, a synthetic androgen. Various concentrations of brefeldin A (BFA, Sigma, MO, USA) dissolved in RPMI 1640 were added to each plate. At 16 h after the BFA treatment, both supernatants and cells were collected and prepared as above for Western analysis.

### Western blot analysis

Whole-cell protein extracts and supernatants were prepared and quantified using DC assay or Bradford assay (BioRad, CA, USA). Equivalent protein samples were loaded into a precast 4–12% NuPage gel (SDS–PAGE), followed by electrophoresis and subsequent transfer onto a nitrocellulose membrane. Ponceau S staining was performed for total protein staining. The membrane was blocked overnight at 4°C with 5% milk in PBST (phosphate buffer solution with 1% Tween 20) and washed five times, 5 min each with PBST. This was followed by incubating the membrane at room temperature with either inducible Hsp70 primary antibody (StressGen, Canada) at 1 : 5000 dilution in PBST or prostate-specific antigen (PSA, Dako, CA, USA) at 1 : 2000 dilution. After an hour of incubation with the indicated antibodies, the membrane was washed as above, followed by a second anti-rabbit/mouse horseradish peroxidase antibody (1 : 100 000) incubation for an additional hour. Protein detection was performed with SuperDura chemiluminescence reagent as per the manufacturer's instructions (Pierce, IL, USA) and visualised with a digital camera. For quantitation, the various bands were analysed with AlphaEaseFC Software version 3.1 (Alpha Innotech Corporation, San Leandro, CA, USA).

### Animal studies

All studies were approved by The Mayo Foundation Institutional Animal Care and Use Committee. Male C57BL/6 mice, 5–6 weeks of age were obtained from Jackson Laboratory and housed in the Mayo Animal Resources Facilities under controlled temperature, humidity, and a 12 h light and dark cycle with food and water *at libitum* in a virus-free environment. Eight mice per group were used for each study. TRAMP-C2 cells were transfected with either pcDNA3.1+Hsp70 or empty vector and collected 24 h post-transfection as described, and irradiated (10 000 rads) and injected subcutaneously. Three separate injections were performed 3 days apart. Each mouse received 1 × 10^6^ cells per injection. At 10 days after the last injection, mice were challenged with 3 × 10^6^ wild-type TRAMP-C2 cells on the opposite flank. In the second study, stably transfected TRAMP-C2 cells were collected, irradiated (10 000 rads), and injected into mice as above. At 10 days after the last injection, mice were challenged with 3 × 10^6^ wild-type TRAMP-C2 cells on the opposite flank. Animals were examined and tumours were measured in three dimensions every other day using a caliper. Tumour volume was calculated, *V*=(length)(width)(depth). Animals were removed from the study when tumour diameter was greater than 1 cm.

### Statistics

Data from the animal studies were analysed by log-rank test or Wilcoxon's signed-rank test as described in the figures. *P*-values <0.05 were considered to be statistically significant.

## RESULTS

### Forced overexpression of Hsp70 can increase Hsp70 secretion

**Figure 1:**
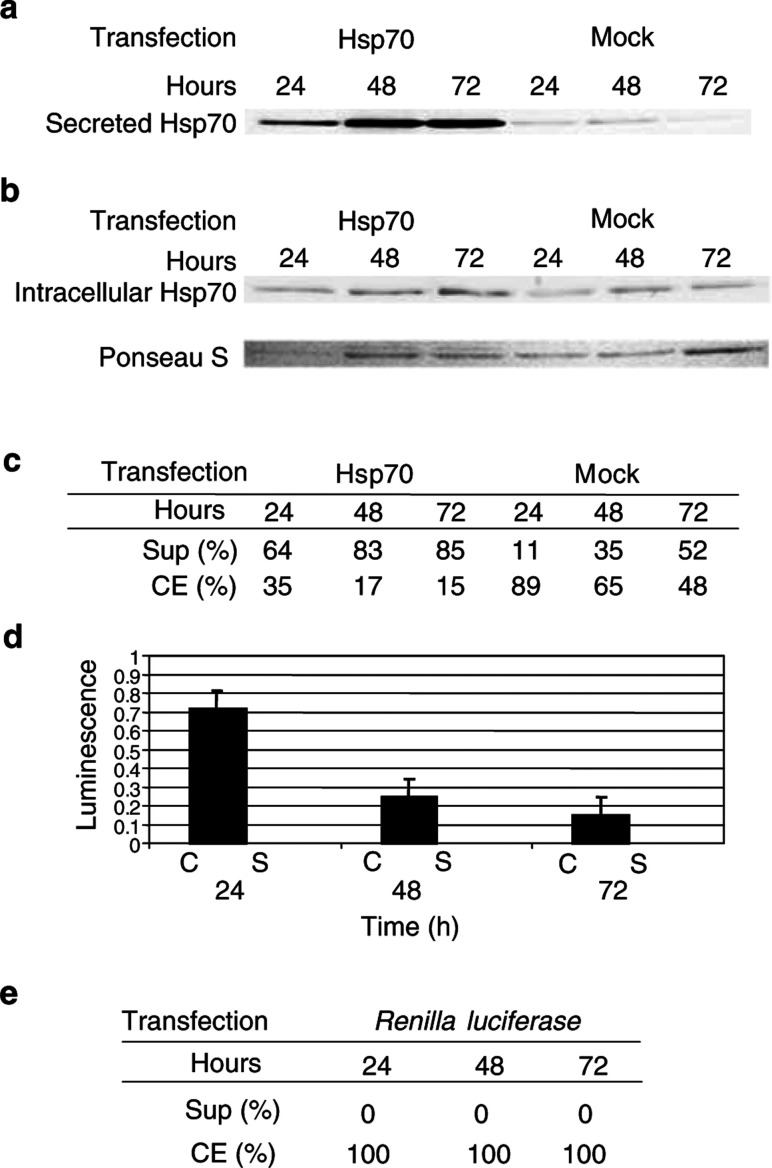
(**A**) Western analysis for Hsp70 in supernatants of pcDNA3.1+Hsp70 and *Renilla luciferase* or mock-transfected TRAMP-C2 cells. Spent media were collected at 24, 48, and 72 h and concentrated as in Materials and methods. (**B**) Western analysis for Hsp70 in cell extracts of Hsp70, *Renilla luciferase*, and mock-transfected TRAMP-C2 cells. Ponseau S was used for normalisation. (**C**) Percent of Hsp70 in supernatants (Sup) and cell extracts (CE) of Hsp70 and mock-transfected TRAMP-C2 cells as determined by densitometry. (**D**). Comparison of luciferase activity in cell extracts (C) *vs* supernatants (S) in C2 cells transfected with pcDNA3.1+Hsp70 and *Renilla luciferase* at various time points. Spent media and whole-cell protein extracts were prepared as above. (**E**) Percentage of renilla protein was determined by luminescence.

During our studies on hsp's, we observed that Hsp70 is present in the routine culture media of TRAMP-C2 cells (data not shown). To test if the presence of Hsp70 in the spent media is a dynamic process and if overexpression can increase its secretion, we transiently transfected TRAMP-C2 cells with a vector coding for murine Hsp70 (pcDNA3.1+Hsp70). As shown in Figure 1

, Hsp70 is detected in both the spent media and the cytosol of transfected and mock-transfected cells. However, increased levels of Hsp70 were noted only in the spent media of transfected cells, while intracellular Hsp70 remained relatively constant over time (Figure 1a and b). The relative amounts of Hsp70 in the supernatants and cell extracts were calculated, and greater percentages of Hsp70 were noted in the supernatants of the transfected samples (24, 48, and 72 h) when compared to the mock-transfected samples (Figure 1c).

### Hsp70 in supernatant is not due to cell leakage

To eliminate the possibility of nonspecific cell leakage by physical damage, TRAMP-C2 cells from the above experiment were cotransfected with pcDNA3.1+Hsp70 and a vector containing the cytoplasmic *Renilla luciferase* as a reporter. Luminescence was used to quantitate the relative amount of renilla protein in the supernatants and cell extracts. No luciferase enzyme activity was detected in the supernatants at any time points (Figure 1d). In addition, results were adjusted to account for concentrated supernatants and represented in percentages (Figure 1e).

### Hsp70 secretion can be found in other human prostate cell lines

**Figure 2:**
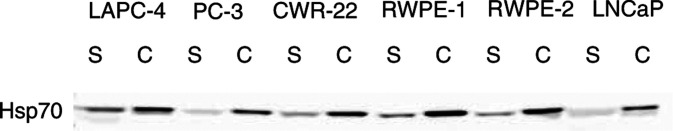
Western analysis for Hsp70 in supernatants (S) and cell extracts (C) of various prostate cell lines. Whole-cell protein extracts and spent media were collected at 48 h after being plated and subjected to Western analysis. Proteins were quantified with Bradford assay and equivalent amounts of proteins were loaded onto SDS–PAGE gel.

Our findings in TRAMP-C2 cells raised the question as to whether Hsp70 secretion is occurring in other prostate cell lines. We examined the spent media of various spontaneous prostate adenocarcinoma cell lines, including LAPC-4, PC-3, CWR-22, and LNCaP cells, and two additional transformed human prostate cell lines, RWPE-1 and RWPE-2. Note that RWPE-1 is not tumorigenic in athymic mice. RWPE-2, derived from RWPE-1, further transformed by Ki-Ras oncogene, is tumorigenic. Western analysis of these human prostate cell lines incubated under routine cell culture conditions was positive for Hsp70 in the supernatants and cell extracts, strongly suggesting that Hsp70 is secreted (Figure 2

).

### Hsp70 secretion is not blocked by a secretion inhibitor, BFA

**Figure 3:**
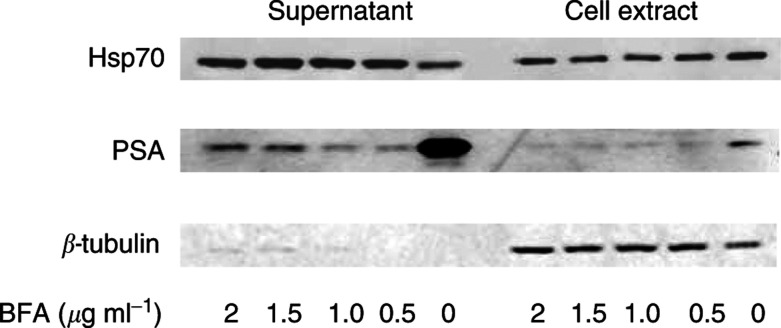
Western analysis for Hsp70, PSA and *β*-tubulin in supernatant and cell extract of LNCaP cells. Whole-cell protein extracts and spent media were prepared 16 h after treatment with various concentrations of BFA (*μ*g ml^−1^). Protein concentrations were quantified and equal amounts of proteins were loaded onto a single gel.

To determine if the observed secreted Hsp70 was through the classical secretory pathway, BFA was used to study the secretion of Hsp70 and PSA in LNCaP cells. At 16 h after treatment, supernatants and cell extracts were collected as above. Western analysis for Hsp70 was performed with PSA as a positive control. LNCaP cells provided a useful model because they are an androgen-responsive human prostate adenocarcinoma cell line that expresses androgen-inducible genes such as PSA (Murtha *et al*, 1993). Prostate-specific antigen is a classical secretory protein that has been well studied, and its secretion and production have been shown to be inhibited by BFA (Gau *et al*, 1997; Konno *et al*, 1998). As shown in Figure 3

, Hsp70 was detected in both supernatants and cell extracts and is not decreased with the addition of BFA, while a decrease in PSA secretion was noted at a concentration of 0.5 *μ*g ml^−1^ BFA or higher. The presence of tubulin in the cell extracts but to only a small extent in the supernatants indicates that the Hsp70 detected in the supernatants is not due to cell death and lysis.

### Forced overexpression of Hsp70 from TRAMP-C2 cells delays tumour growth and extends survival of C57BL/6 male mice

#### Transient overexpression experiment

**Figure 4:**
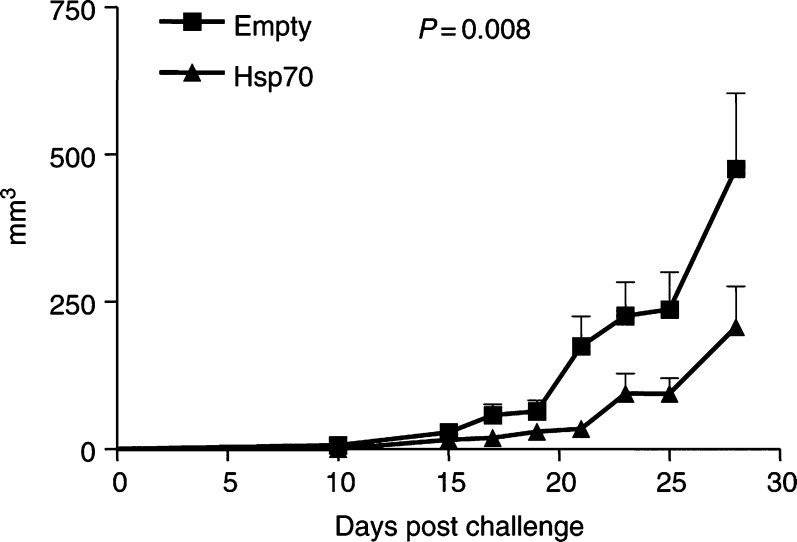
TRAMP-C2 tumour growth rate in C57BL/6 male mice after injecting with transiently transfected TRAMP-C2 cells with Hsp70 or empty vector. Tumours were measured every other day. *P*-values of <0.05 were considered statistically significant.

**Figure 5:**
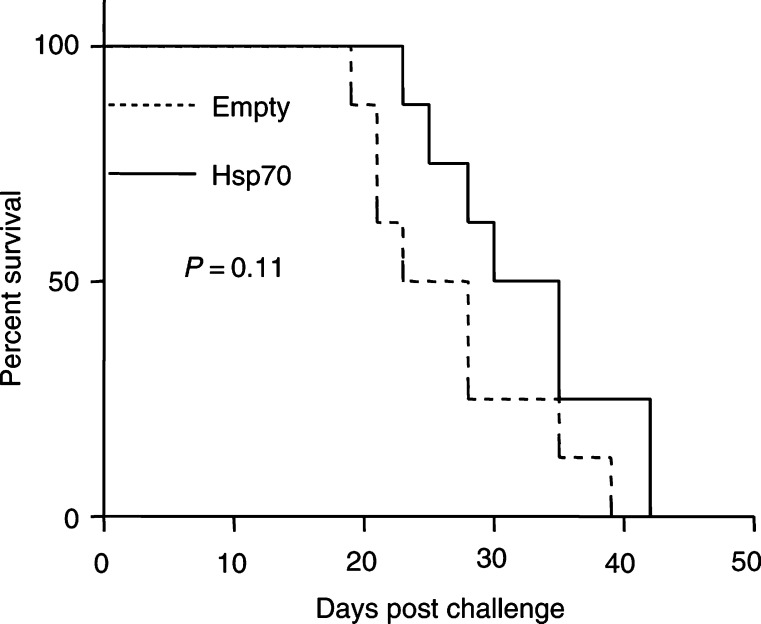
Percent survival of mice with transiently transfected Hsp70 TRAMP-C2 cells or empty vector.

To test whether forced oversecretion of Hsp70 from prostate cancer cells can provide protection from tumour growth *in vivo*, TRAMP-C2 cells were transiently transfected with pcDNA3.1+Hsp70 and injected subcutaneously into syngeneic C57BL/6 male mice. Cells transfected with empty vector were used as a control. At 10 days after last injection, mice were challenged with nontransfected TRAMP-C2 cells on the opposite flank. As shown in Figure 4

, there is a delay in TRAMP-C2 cell growth in mice previously inoculated with Hsp70-expressing C2 cells. Statistical significance was observed at *P*=0.008 as analysed by Wilcoxon's signed-rank test between the groups (Figure 4). We also examined survival as defined by the time until the diameter of the tumour was greater than 1 cm. We found that there was a difference in the survival rates between the two groups that was not statistically significant (Figure 5

, *P*=0.11). This experiment suggests a protective effect offered by inoculation with cells forced to overexpress Hsp70.

### Stable overexpression experiment

**Figure 6:**
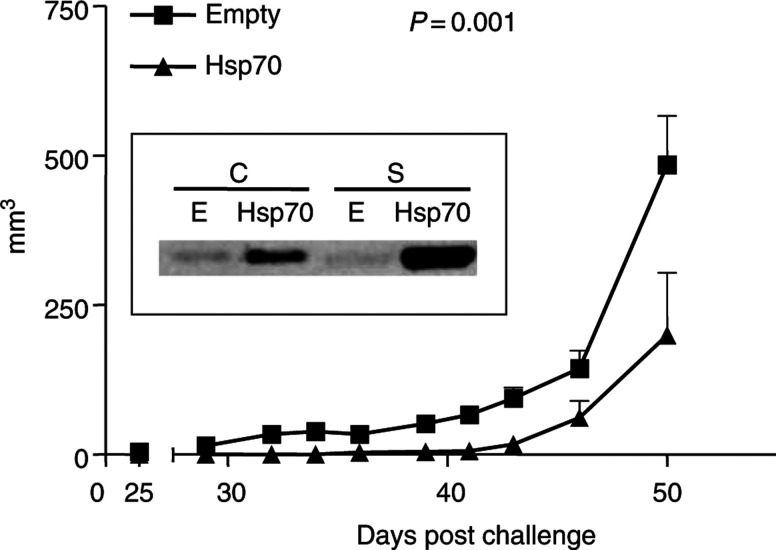
TRAMP-C2 tumour growth rate in C57BL/6 male mice after injecting with stably transfected Hsp70 TRAMP-C2 cells. Tumours were measured every other day. *P*-values of <0.05 were considered statistically significant. Inlet: Western analysis of Hsp70 in pcDNA3.1+murine Hsp70 (Hsp70)- and pcDNA3.1 (E)-transfected stable clones. Cell extracts (C) and supernatants (S).

**Figure 7:**
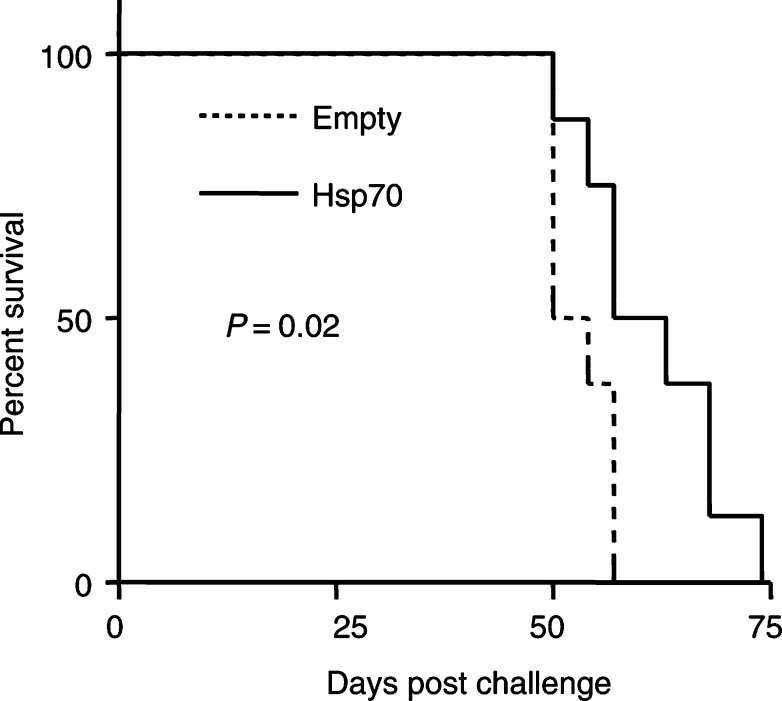
Percent survival of mice with stably transfected Hsp70 TRAMP-C2 cells or empty vector.

Furthermore, stable TRAMP-C2 transfectants with pcDNA3.1+Hsp70 or empty vector as a control were used to reproduce the above experiment. Western analysis of these clones verified an increase in intra- and extracellular Hsp70. Injections with stable clones and subsequent live, nontransfected TRAMP-C2 challenge were performed as above. As shown in Figure 6

, there was a statistically significant delay in TRAMP-C2 tumour growth in mice previously injected with Hsp70-expressing stable clones (*P*=0.001). In addition, in this experiment there was a significant difference in survival between the two study groups (Figure 7

, *P*=0.02).

## DISCUSSION

In the course of extending our previous studies on hsp's in prostate cancer, we found that Hsp70 is secreted into the spent media by some prostate cell lines under routine cell culture conditions, although a rigorous examination of all the cell lines remains to be completed. The significance of *in vivo* secretion of Hsp70 remains to be further elucidated. Previous studies have shown the release of hsp's into the cultured media by rat and chick embryo cells, squid glial cells, and yeast following heat shock (Hightower and Guidon, 1989; Russo *et al*, 1992; Guzhova *et al*, 2001). It is believed that these hsp's are important in cell proliferation during embryo morphogenesis, in addition to acting as protective factors for the surrounding cells in the presence of environmental stress (Hightower and Guidon, 1989; Russo *et al*, 1992; Guzhova *et al*, 2001). Our extensive literature search indicates that this is the first time that the Hsp70 secretion is documented in mouse and human prostate cancer cells. In addition, Hsp70 secretion can be increased with overexpression. This raised an interesting implication in that this oversecretion might have the potential to be utilised in generating antitumour immunity.

Hsp studies have attempted to elucidate the means by which intracellular hsp's can interact with extracellular immune cells. One of the possibilities is that hsp's are released during tumour cell necrosis, leading to the induction of immune response. Studies by Melcher *et al*, utilising the suicide gene transfer system, herpes simplex virus thymidine kinase/gancyclovir (HSVtk/GCV), noted different patterns of cell death in various tumour cells. Herpes simplex virus thymidine kinase/gancyclovir utilises the strategy in which a gene coding for a prodrug-converting enzyme is delivered into tumour cells, followed by the administration of the prodrug. Thus, the enzyme converts the prodrug into a toxic compound that kills the cells (Vile *et al*, 1997). In this particular study, cells that became necrotic with HSVtk/GCV treatments were found to express higher levels of hsp's mRNA when compared to cells that were apoptotic. Follow-up *in vivo* studies showed a decrease in tumorigenicity of hsp-transfected cells (Melcher *et al*, 1998). Studies subjecting tumour cells to rapid freeze–thaw cyles to mimic necrosis also noted an increase in hsp's in the cell lysates and supernatants, with corresponding decrease in tumorigenicity. Studies also supported cell surface expression as an avenue by which hsp's can interact with extracellular immune cells. Studies utilising membrane-bound hsp constructs noted an increase in immunogenicity of transfected cells (Wu *et al*, 1999; Chen *et al*, 2002). Recently, a study demonstrated a decrease in tumorigenicity of hsp110-overexpressing colon cancer cells (Wang *et al*, 2002). Further studies should clarify the role each of these mechanisms has on immunogenicity.

Although Hsp70 has been regarded as an intracellular protein, we found its presence in the extracellular media despite the addition of BFA, a reversible inhibitor that blocks protein translocation at the level of the endoplasmic reticulum–Golgi juncture and the trans-Golgi network (Schatz and Dobberstein, 1996; Cleves, 1997). Our findings indicate that these prostate cancer cells secrete Hsp70 via a mechanism other than the well-studied classic vesicular secretory pathway. This ‘nonclassical’ secretion of proteins that lack a typical N-terminal signal peptide has been observed in several other proteins such as fibroblast growth factors 1 and 2, interleukin-1, and viral proteins (herpes simplex tegument proteins) (Cleves, 1997). The fact that Hsp70 is released into the culture media by prostate cells without known stressors, coupled with its anticancer activity raised some interesting questions: first, whether hsp's are secreted *in vivo* by prostate cancer cells, second and more importantly, how does this phenomena fit into the evolution of host tolerance to cancer cells.

In order to test our hypothesis that Hsp70 oversecretion from prostate cancer cells can potentially be utilised as an anticancer agent, murine Hsp70 was overexpressed in TRAMP-C2 cells and tested *in vivo*. TRAMP-C2 cells, a transplantable murine epithelial prostate cancer cell line, provide a useful model for the study of prostate cancer therapies (Greenberg *et al*, 1995). Our study showed that there is a significant decrease in the tumorigenicity of TRAMP-C2 cells in mice injected with TRAMP-C2 cells oversecreting Hsp70, in addition to a significant difference in survival between mice injected with Hsp70 oversecreting cells and control.

We can speculate from our *in vitro* results and previous studies that an undefined level of Hsp70 extracellularly might be involved in cancer cell protection. This is in concordance with correlation studies that suggest hsp's as unfavourable prognostic factors for progression in some types of cancer (Nylandsted *et al*, 2000a,2000b; Lebret *et al*, 2003). Moreover, studies in our laboratory and others have shown that increased hsp's induces antitumour activities. A study by Podack and co-workers who constructed a gp96-Ig fusion protein noted an increase in tumour immunogencity in cells transfected with this construct, suggesting that increased hsp secretion can act as a stimulatory signal for the breaking of host immune tolerance (Yamazaki *et al*, 1999). Additional studies of hsp secretion will likely offer insights and help us to answer fundamental immunologic questions with respect to the development of tolerance and immunity.
